# Recurrent Nonbacterial Thrombotic Endocarditis after Vegetectomy of Bioprosthetic Mitral Valve

**DOI:** 10.1093/icvts/ivaf192

**Published:** 2025-08-19

**Authors:** Taro Nakazato, Matsuda Yasuhiro, Tatsuya Ozaki, Mutsunori Kitahara

**Affiliations:** Department of Cardiovascular Surgery, Kansai Rosai Hospital, Amagasaki, 660-8511, Japan; Department of Cardiology, Kansai Rosai Hospital, Amagasaki, 660-8511, Japan; Department of Cardiovascular Surgery, Kansai Rosai Hospital, Amagasaki, 660-8511, Japan; Department of Cardiovascular Surgery, Kansai Rosai Hospital, Amagasaki, 660-8511, Japan

**Keywords:** nonbacterial thrombotic endocarditis, mitral valve replacement, vegetectomy, papilloma

## Abstract

We report a case of recurrent nonbacterial thrombotic endocarditis (NBTE) following vegetectomy of bioprosthesis without malignancy or autoimmune disorders. A 76-year-old woman underwent mitral valve replacement with a bioprosthesis for mitral regurgitation. Six years later, an outpatient echocardiography revealed a mobile vegetation incidentally. No signs of infection were observed, and various tests ruled out malignancy or autoimmune disorders. Pathological findings after an urgent vegetectomy also showed no signs of infection, and the diagnosis was NBTE. Anticoagulation therapy with warfarin was continued, but 1 year after surgery, an outpatient echocardiography revealed NBTE recurrence. After the patient underwent anticoagulation therapy with heparin and resection of the papilloma of the maxillary sinus, the vegetation disappeared, and there has been no recurrence. Since NBTE can recur, strict lifelong follow-up with anticoagulation therapy might be required.

## INTRODUCTION

Nonbacterial thrombotic endocarditis (NBTE) is a rare condition in which vegetations attach to heart valves without inflammation or bacteria; NBTE is often caused by malignancy or autoimmune disorders.^1^ There is no consensus on treatment, but anticoagulant therapy and treatment for primary disease are often selected, and surgical treatment might also be performed.^1^ Here, we report a very rare case of recurrent NBTE after vegetectomy of bioprosthetic mitral valve without malignancy or autoimmune disorders.

## CASE PRESENTATION

**Figure 1.**
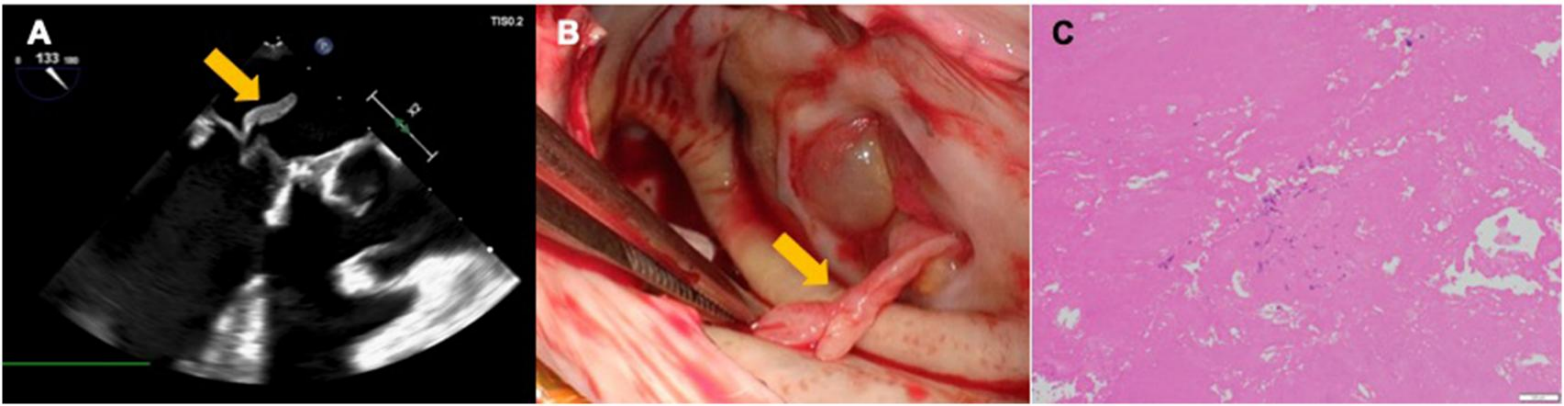
Transoesophageal Echocardiography Showing the Mobile Vegetation Attached to Bioprosthesis (A). Intraoperative Endoscopy Showing Sterile Vegetation Attached to the Bioprosthesis (B). Hematoxylin-Eosin Staining Showing Almost no Cellular Components within the Vegetation (C)

**Figure 2.**
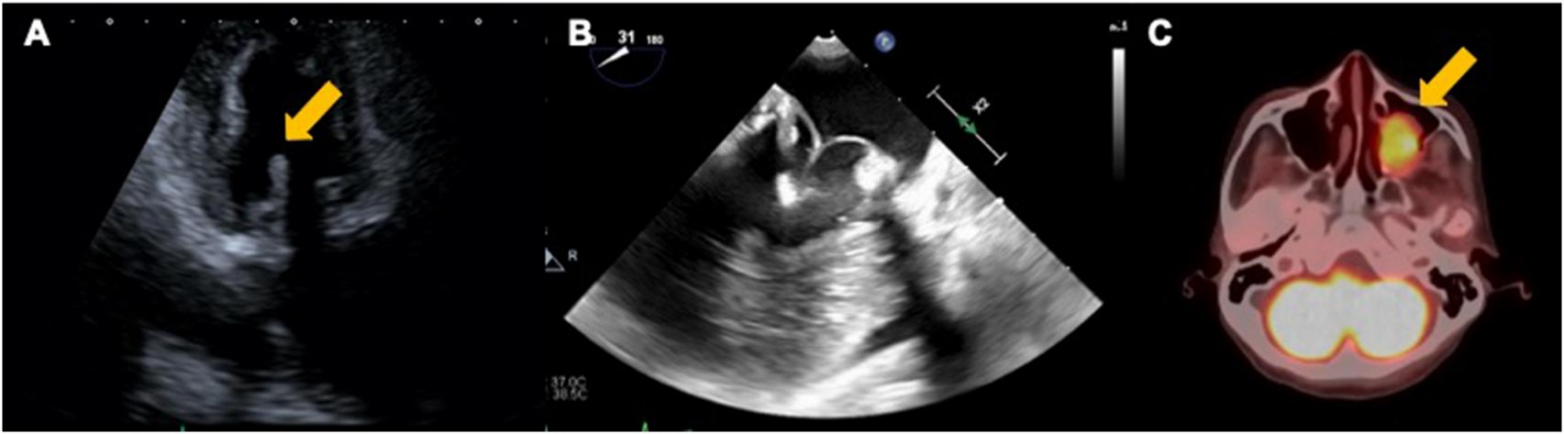
Transthoracic Echocardiography Showing Recurrent NBTE with the Mobile Vegetation Attached to Bioprosthesis (A). Transoesophageal Echocardiography Showing the Disappearance of Vegetation after Anticoagulant Therapy (B). Positron Emission Tomography-Computed Tomography Showing Increased Uptake of Fluorodeoxyglucose in the Right Maxillary Sinus (C)

A 76-year-old woman had undergone mitral valve replacement (MVR) with a 29-mm bioprosthetic valve to treat mitral regurgitation 6 years prior. An outpatient echocardiography revealed mobile vegetation (10 mm × 7 mm) attached to the bioprosthesis, which had not been detected in the previous year’s test (**Figure 1A** and **Video 1**). She was afebrile, and her laboratory testing showed no inflammatory changes. After admission, blood cultures were negative. Chest and abdominal CT revealed no obvious tumours, and additional blood tests ruled out autoimmune disorders. Urgent vegetectomy or re-MVR was planned to prevent embolic events. Intraoperative surgical findings showed that sterile vegetation was attached to the bioprosthesis (**Figure 1B**). Since there were no signs of infection in the bioprosthesis or surrounding tissues and no deterioration of the bioprosthesis, vegetectomy was performed. Pathological findings showed that the vegetation was fibrin thrombi, and almost no cellular components were observed (**Figure 1C**). Postoperative course was uneventful, and the patient was discharged after anticoagulation with warfarin. The outpatient course was initially uneventful, with warfarin control at a prothrombin time-international normalized ratio (PT-INR) of around 2, and outpatient echocardiography showed no recurrence 6 months after vegetectomy. One year after vegetectomy, follow-up echocardiography revealed vegetation attached to the prosthetic valve (**Figure 2A** and **Video 2**). As in the previous hospitalization, she had no fever; there were no inflammatory changes or hypercoagulable conditions in the laboratory testing, and NBTE recurrence was suspected. We selected intensive anticoagulant therapy with heparin after readmission because the vegetation was not mobile, and CT did not reveal any obvious embolism. Heparin dose was adjusted to maintain an activated partial thromboplastin time (APTT) of ≥80 seconds. A follow-up echocardiography 3 weeks after anticoagulant therapy showed that the vegetation had disappeared (**Figure 2B** and **Video 3**). PET-CT showed an increased uptake of fluorodeoxyglucose in the tumour in the right maxillary sinus (max. SUV 8.77) (**Figure 2C**). Although the tumour was suspected to be benign, a maxillary sinus tumour resection was also performed on the fourth day after admission, as this tumour might be the cause of NBTE. Pathological findings showed that this tumour was papilloma, and no malignant findings were observed. Anticoagulant therapy was changed from heparin to warfarin after confirming the disappearance of vegetations, and the patient was discharged with PT-INR controlled at ≥2. The patient is being followed up as an outpatient, and no recurrence has been noted to date.

## DISCUSSION

Nonbacterial thrombotic endocarditis pathogenesis is still unclear, but factors related to NBTE have been reported to include immune complexes, hypoxia, hypercoagulability, and carcinomatosis.^1^ Conversely, few reports described NBTE occurring in patients without any particular disease or after valve replacement.^2^^,^^3^ Here, NBTE was detected incidentally without any symptoms after bioprosthetic valve replacement in a patient without underlying malignancy or autoimmune disorders. Moreover, this case is considered extremely rare in that there have been no previous reports of recurrent NBTE 1 year after anticoagulant therapy following vegetectomy. There have been no reports of vegetectomy for NBTE; however, some case reports of thrombectomy following bioprosthetic valve replacement, including our case, indicate that no recurrence of thrombosis was observed.^4^^,^^5^ Nonbacterial thrombotic endocarditis treatment is based on correction of the underlying disease; however, it is quite difficult in our case without an underlying disease. After vegetectomy, sufficient anticoagulant therapy with warfarin was performed without a decrease in PT-INR during the outpatient clinic, but NBTE recurred. During the second hospitalization, anticoagulant therapy with heparin was performed, and the tumour was resected to correct a possible underlying disease. In Japan, since low molecular weight heparin cannot be prescribed in an outpatient clinic, strict warfarin control has been performed by prolonging the PT-INR at ≥2, and there has been no NBTE recurrence. Although no association between papilloma and NBTE has been reported to date, it is crucial to correct the underlying disease as much as possible, and papilloma resection might have contributed to no recurrence of NBTE in our case. Strict life-long follow-up is required, keeping in mind the possibility of recurrence.

## Funding

None declared.

## CONFLICTS OF INTEREST

None declared.

## Data Availability

The data underlying this article cannot be shared publicly due to the privacy of individuals who participated in the study. The data will be shared on a reasonable request to the corresponding author.
